# Case Report: Pregnancy complicated with pulmonary arteriovenous malformation—diagnosis and surgical management

**DOI:** 10.3389/fsurg.2025.1647557

**Published:** 2025-10-08

**Authors:** Wenying Ji, Mingju Fu, Hongquan wang, Zhifen He, Junhao Chen, Shi Fu, Yuanzhi Fu, Xingcheng Zhu

**Affiliations:** 1 The Department of Obstetrics, Qujing Second People’s Hospital, Qujing, China; 2 Department of Urology, The Second Affiliated Hospital of Kunming Medical University, Kunming, China; 3 School of Clinical Medicine, Kunming University of Science and Technology, Kunming, Yunnan, China; 4 Department of Clinical Laboratory, The Second People’s Hospital of Qujing City, Qujing, Yunnan, China

**Keywords:** pulmonary arteriovenous malformation (PAVM), pregnancy complications, transcatheter embolization, critical care, multidisciplinary team

## Abstract

Pulmonary arteriovenous malformation (PAVM) during pregnancy is a rare but life-threatening condition, exacerbated by gestational hemodynamic changes. This case report describes a 24-year-old gravida at 27 + 6 weeks presenting with recurrent hemoptysis and hypoxemia. Contrast-enhanced transthoracic echocardiography (TTCE) and computed tomography angiography (CTA) confirmed a left upper lobe PAVM. A multidisciplinary team prioritized cesarean delivery under combined spinal-epidural anesthesia to minimize hemodynamic instability, followed by staged transcatheter embolization (TCE) postpartum—marking the first TCE application for PAVM in Yunnan, China. The neonate (1,100 g) demonstrated favorable Apgar scores, and maternal recovery was achieved without recurrence. Key challenges included balancing fetal safety with urgent intervention, optimizing perioperative critical care, and addressing risks of hypoxemia and hemorrhage. This case underscores the efficacy of coordinated obstetrics, anesthesiology, and interventional radiology teams in managing high-risk pregnancies with PAVM. It highlights spinal-epidural anesthesia for hemodynamic stability, TCE as a minimally invasive solution, and the necessity of long-term surveillance for recurrence. These insights advance clinical strategies for rare cardiopulmonary-obstetric emergencies, emphasizing tailored interventions and multidisciplinary collaboration to ensure maternal-fetal safety.

## Introduction

Pulmonary arteriovenous malformation (PAVM) is a rare vascular anomaly of the lung, characterized by one or more abnormal direct connections between pulmonary arteries and veins, forming arteriovenous fistulas. PAVMs cause abnormal blood flow, allowing some blood to bypass the alveolar capillaries and enter pulmonary veins without oxygenation. This allows deoxygenated blood to enter systemic circulation, leading to hypoxemia. Clinically, this can manifest as a range of hypoxia-related symptoms, including dyspnea, fatigue, dizziness, and even syncope. In severe cases, it may lead to serious complications such as cerebral thrombosis or ischemia of vital organs (1). Surgical intervention is one of the treatment options for patients with PAVM. It involves the resection of affected lung tissue or abnormal vessels to restore normal blood flow, reduce right-to-left shunting, improve oxygenation, and alleviate clinical symptoms. Although surgery can effectively remove overt lesions, it may not eliminate all abnormal vasculature due to the potential for multifocal or complex vascular malformations. Small or occult lesions are particularly difficult to detect and completely excise, thereby increasing the risk of recurrence (2). Although surgery can relieve certain symptoms, the occurrence of recurrent PAVMs necessitates further treatment, indicating that the effectiveness of surgical intervention remains limited. During pregnancy, PAVMs become more challenging due to increased blood volume and cardiac output, including increased blood volume, elevated cardiac output, and venous dilation. These changes can predispose patients to serious complications such as thromboembolism and cerebral abscesses, significantly complicating treatment. Meanwhile, symptoms of PAVM such as hypoxemia or dyspnea may be similar to normal pregnancy-related changes, thereby delaying diagnosis and complifying timely management (3).

## Case report

**Figure 1:**
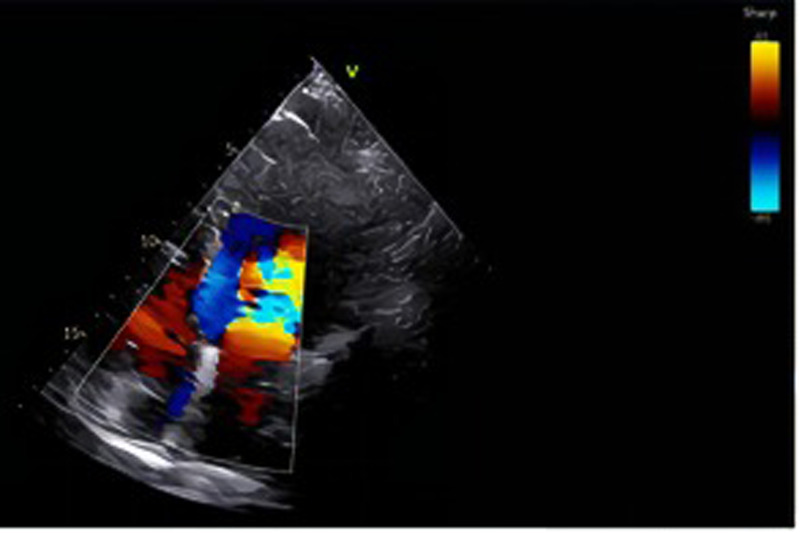
In the lower left to mid region of the image, a distinct mosaic flow signal comprising red, blue, and yellow is observed, located near the posterior wall of the atrium or the pulmonary vein inflow area. Under physiological conditions, color Doppler signals are typically confined to the valvular orifice and adjacent flow regions. However, in this case, the jet deviates from the valvular axis and traverses obliquely upward, suggesting the presence of an abnormal flow pathway from a non-physiological direction. This imaging feature raises suspicion of a pulmonary arteriovenous malformation (PAVM), which may facilitate a right-to-left shunt.

**Figure 2:**
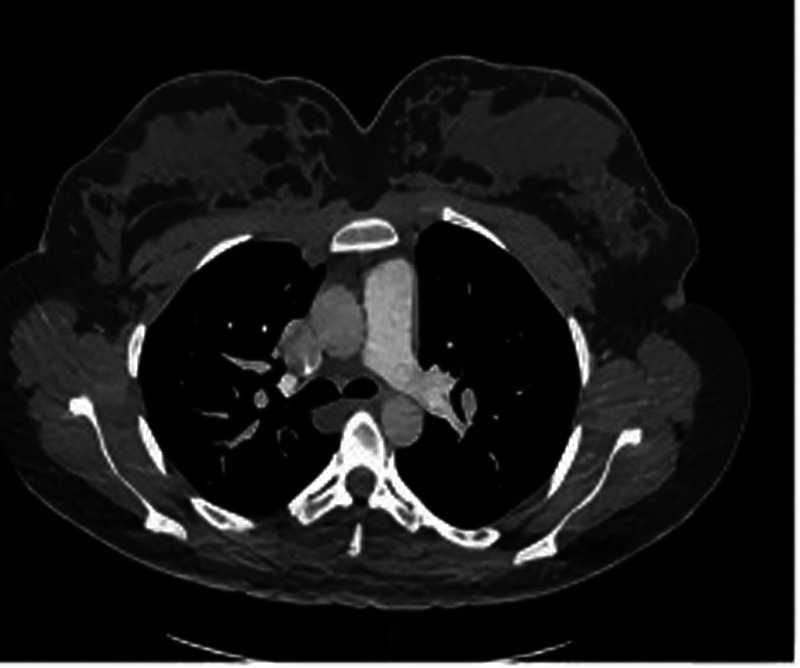
One or more enlarged, tortuous soft-tissue density structures are observed traversing the pulmonary parenchyma in the right hilar region (left side of the image). These structures demonstrate an atypical course and morphology inconsistent with normal pulmonary arteries or veins, strongly suggestive of a pulmonary arteriovenous fistula (PAVF).

A 24-year-old woman was admitted at 27 + 6 weeks of gestation due to recurrent hemoptysis. She reported a sudden onset of coughing during sleep without any identifiable trigger, followed by expectoration of approximately 20 ml of bright red blood. The episode was accompanied by orthopnea, but there were no symptoms of chest pain, dyspnea, or fever. The patient had no significant past medical history, including no hypertension, coronary artery disease, diabetes mellitus, hyperthyroidism, or exposure to toxins or radiation. On admission, physical examination revealed bilateral lower limb edema. Her vital signs were: temperature 37.4 ℃, heart rate 126 bpm, respiratory rate 20 /min, blood pressure 120/70 mmHg, and oxygen saturation 93% under oxygen therapy (Oxygen inhalation state). Transthoracic echocardiography with contrast revealed a positive right heart contrast study, suggesting the presence of a PAVM (Figure 1). Subsequent contrast-enhanced pulmonary artery CT further confirmed the diagnosis, indicating a pulmonary arteriovenous malformation in the upper lobe of the left lung, suggesting by localized pulmonary atelectasis (Figure 2). A multidisciplinary consultation was conducted. Based on the patient's history, physical findings, and auxiliary tests, the final diagnoses were: pregnancy complicated with pulmonary hemorrhage, pulmonary arteriovenous fistula, pulmonary infection, congenital pulmonary vascular malformation, pregnancy with hypothyroidism, type I respiratory failure, pregnancy with sinus tachycardia, hypokalemia, and respiratory alkalosis.

Six days later (April 22, 2023), the patient still had chest tightness and orthopnea, with one further episode of hemoptysis (About 13 ml). Vital signs were: temperature 36.1 ℃, heart rate 112 bpm, respiratory rate 20 /min, blood pressure 118/60 mmHg. Given repeated hemoptysis and worsening maternal condition, a cesarean section was scheduled. On April 23, 2023, after obtaining informed consent from the patient and her family, a cesarean section was successfully. A live female infant in cephalic presentation was delivered, weighing 1,100 g, with no external malformations. The initial Apgar score was 9; Apgar scores at 1 and 5 min were 6 and 9, respectively. The newborn was stabilized by the neonatal team. During the procedure, approximately 100 ml of amniotic fluid with I° turbidity was aspirated. The umbilical cord was 30 cm in length. The umbilical cord was 30 cm in length. Due to ongoing intrauterine bleeding, a sterile gauze tampon was placed in the uterine cavity for removal after 24 h. Estimated intraoperative blood loss was approximately 400 ml. The operation was completed uneventfully. Postoperatively (April 24, 2023), her condition improved markedly: temperature 36.5 ℃, heart rate 85 bpm, respiratory rate 19 /min, blood pressure 100/59 mmHg, SpO_2_ 98% under oxygen. She reported no chest tightness, no orthopnea, and no hemoptysis, and could lie in the left lateral position comfortably. At discharge (May 2, 2023), vital signs were stable without oxygen therapy: temperature 36.3 ℃, heart rate 78 bpm, respiratory rate 19 /min, and SpO_2_ 95%. Laboratory results, including complete blood count and biochemical tests, were within normal limits. And discharged from the hospital smoothlyOne month postpartum, under the guidance of the interventional radiology team, the patient underwent the first transcatheter embolization of the pulmonary arteriovenous fistula. A second embolization was performed one year later. The patient has since fully recovered. The detailed comparison of pre- and post-treatment images can be found in Supplementary Material S1, which showcases significant changes and helps assess the therapeutic effect.

## Discussion

Pulmonary arteriovenous malformation (PAVM) during pregnancy is a rare pulmonary vascular disorder, typically characterized by abnormal connections between arteries and veins that bypass the pulmonary capillary network. PAVMs can lead to inadequate oxygenation, resulting in clinical manifestations such as hypoxemia, cyanosis, and dyspnea, posing potential threats to both maternal and fetal health. During pregnancy, the increase in blood volume and changes in cardiac output may exacerbate the symptoms of PAVM, thereby increasing the risk of maternal and fetal complications. For example, rupture of a PAVM can result in massive hemorrhage, potentially leading to shock or even maternal death.

The diagnosis of PAVM during pregnancy is challenging due to symptom overlap with normal pregnancy changes, such as shortness of breath, fatigue, and hypoxemia. These symptoms can delay recognition, and imaging modalities like CT or pulmonary angiography, though necessary for diagnosis, pose fetal risks due to radiation exposure. Misdiagnosis is also common, as PAVM can be mistaken for conditions like pulmonary embolism (4). Clinicians must balance diagnostic accuracy with fetal safety when choosing imaging methods. If PAVM leads to complications such as pulmonary hemorrhage or hemothorax, surgical intervention may be required, though surgery during late pregnancy carries significant risks for both the mother and fetus. Interventional therapy, such as transcatheter embolization (TCE), has been shown to be effective for PAVM patients. However, performing TCE during pregnancy requires extreme caution. It is generally recommended to carry out the procedure during the second or third trimester to minimize fetal radiation exposure. The timing of intervention is critical; performing it too early or too late may compromise both therapeutic efficacy and maternal-fetal safety. Physicians must not only be familiar with the indications and risks of various treatment modalities but also tailor an individualized treatment plan based on gestational age and patient-specific factors. Precise control of the vascular intervention is essential to ensure complete embolization of the PAVM while avoiding injury to surrounding tissues and the fetus.

Despite treatment, there remains a risk of recurrence in pregnant patients with PAVM. Studies have shown that up to 84.4% of embolized PAVMs may recanalize, leading to the return or worsening of symptoms. This underscores the need for long-term follow-up after treatment to monitor for potential complications. Even postpartum, patients require continued surveillance with regular check-ups to ensure there is no recurrence of the condition. This highlights the need for physicians to have strong long-term management capabilities to ensure sustained maternal health (5).

Currently, the diagnosis and treatment of PAVM mainly rely on contrast-enhanced transthoracic echocardiography, CT, and transcatheter embolization therapy. Contrast-enhanced transthoracic echocardiography is the preferred screening test for PAVM (6). It is commonly used to detect intracardiac right-to-left shunts. In conditions such as PAVM, such shunting may allow blood to flow directly from the right atrium or right ventricle to the left atrium or left ventricle without passing through pulmonary oxygenation. Contrast-enhanced transthoracic echocardiography has demonstrated high sensitivity and a low false-negative rate. In one study, its sensitivity reached 92% to 93% (7). It not only effectively identifies most cases of PAVM but also has the capability to detect small malformations. The high reproducibility and low false-negative rate of contrast-enhanced transthoracic echocardiography (TTCE) make it the preferred screening method for PAVM, especially in patient populations where radiation exposure should be avoided. In addition, CT is considered the gold standard for confirming the diagnosis of PAVM (1). CT provides precise anatomical details, including abnormal connections between pulmonary arteries and veins, vascular course, size, and the exact location of the malformation. While TTCE relies primarily on hemodynamic changes to detect right-to-left shunts, CT allows visualization of the spatial distribution and localization of abnormal vessels. In one study evaluating PAVM before and after treatment, CT accurately identified 98.2% of PAVM cases. Compared with conventional pulmonary angiography, CT offers significant advantages in assessing the location, branching, and morphological features of arteriovenous malformations, particularly in clearly delineating vascular pathways and blood flow distribution (8). Although TTCE is highly sensitive to abnormal blood flow patterns, such as shunting, it has limited ability to localize small anatomical changes or complex vascular malformations. CT compensates for this limitation by providing clearer anatomical localization. For treatment, transcatheter embolization therapy is considered the gold standard for managing PAVM (6). It is a widely used treatment for PAVM, particularly in cases with high blood flow or significant clinical symptoms. Transcatheter embolization therapy has demonstrated a high clinical success rate and low incidence of complications in the treatment of PAVM, and has become an important therapeutic option. However, in the management of PAVM during pregnancy, cesarean section may be the preferred initial intervention, particularly in patients with severe complications or high-risk obstetric factors. Acute hemothorax and rupture of PAVM are among the serious complications that may occur during pregnancy. In such cases, cesarean section often plays a crucial role in managing the complication, especially in emergency situations, where successful delivery can facilitate subsequent treatment and recovery. For example, one report described a pregnant woman at 34 + 5 weeks of gestation who experienced massive hemothorax due to PAVM rupture. She underwent emergency cesarean section followed by thoracic drainage and CT examination, and subsequently received successful embolization therapy (9). This case demonstrates that PAVM during pregnancy, if not managed promptly, may lead to severe complications. However, with timely cesarean section and appropriate postoperative treatment, both maternal and fetal outcomes can be effectively managed. In cases where PAVM causes acute complications such as hemothorax, worsening right-to-left shunting, or severe hypoxemia, cesarean delivery can alleviate symptoms and ensure maternal and fetal safety. Following delivery, embolization therapy can be performed to address the underlying PAVM, often resulting in favorable recovery.

Beyond pregnancy-related complications, atypical cases of PAVM also underscore the importance of heightened clinical vigilance in imaging evaluation, surgical decision-making, and long-term follow-up. Topaloglu et al. (10) reported a case involving a 21-year-old female patient who presented with persistent cough and was found to have a deeply located arteriovenous malformation (AVM) in the right lower lobe near the pulmonary hilum. Thoracic computed tomography revealed a 42 × 38 mm well-circumscribed lesion, ultimately confirmed and successfully resected via anatomical lobectomy. Intraoperatively, the lesion was identified as originating from the bronchial artery and draining directly into the pulmonary vein—features indicative of a complex, deep-seated type of PAVM not amenable to percutaneous transcatheter embolization. This case highlights that in instances where the feeding artery is not clearly visualized or the lesion is inaccessible to interventional techniques, surgical resection remains a definitive and curative approach, especially for complex or refractory PAVMs (11).

It is important to note that untreated PAVMs carry a significant risk of progressive enlargement and life-threatening complications such as hemoptysis, hemothorax, or cerebral abscess (3). Although catheter-based embolization has emerged as the first-line therapy in recent years, it may be ineffective in cases with high-flow lesions, deep anatomical locations, or intricate vascular architecture. Therefore, preoperative high-resolution imaging, including CT angiography, and precise intraoperative identification of the malformation are crucial. Treatment strategies should be tailored to individual anatomical and hemodynamic characteristics, with careful consideration of the accessibility and feasibility of intervention.

Despite their rarity, PAVMs exhibit marked clinical heterogeneity and unpredictability. The diversity in pathological types, underlying etiologies, symptom profiles, and therapeutic responses necessitates a personalized approach to management. Future clinical efforts should focus on more refined preoperative classification, individualized selection of surgical or endovascular techniques, and long-term surveillance through imaging and functional assessments, with the goal of reducing recurrence and improving long-term outcomes.

This case has several unique features that merit emphasis. First, PAVM during pregnancy is extremely rare, and presentation with recurrent hemoptysis and hypoxemia in the late second trimester is unusual. The clinical manifestations overlapped with common pregnancy-related cardiopulmonary changes, such as increased blood volume, cardiac output, and venous dilation, making early recognition more difficult. Second, the diagnosis was confirmed using both TTCE and CT, which together allowed for accurate characterization of the lesion while balancing maternal–fetal safety.

From a procedural standpoint, several considerations required careful attention. The decision to prioritize cesarean delivery before definitive embolization allowed stabilization of the fetus and facilitated subsequent interventional management. Timing of embolization postpartum was equally important, as immediate intervention would have carried higher risks of radiation exposure and hemodynamic compromise. Close perioperative monitoring in the ICU, along with careful correction of electrolyte imbalances and optimization of respiratory function, were also crucial for successful outcomes.

The lessons learned from this case can provide practical guidance for similar high-risk pregnancies. Early multidisciplinary collaboration between obstetrics, anesthesiology, interventional radiology, and critical care teams is essential. Individualized planning should account for gestational age, maternal hemodynamic status, and fetal condition. When severe complications arise, cesarean delivery under stable anesthetic conditions can be a life-saving bridge to definitive interventional therapy. Postpartum transcatheter embolization offers a minimally invasive, effective treatment with favorable maternal recovery and low recurrence risk, though long-term surveillance remains necessary.

## Conclusion

Pulmonary arteriovenous malformation (PAVM) during pregnancy is a rare condition. In this case, the patient was diagnosed using contrast-enhanced transthoracic echocardiography (TTCE) and computed tomography (CT), and successfully treated with cesarean section and embolization therapy following severe hemoptysis and hypoxemia. TTCE confirmed the presence of a right-to-left shunt, while CT provided precise anatomical details that guided treatment planning. The cesarean section effectively avoided potential risks associated with vaginal delivery, and embolization successfully occluded the abnormal vessels, improving oxygenation. This case highlights the importance of multidisciplinary collaboration and an integrated treatment approach in the management of PAVM during pregnancy, ensuring maternal and fetal safety and significant symptom relief.

## Data Availability

The original contributions presented in the study are included in the article/Supplementary Material, further inquiries can be directed to the corresponding authors.
