# Supplementary material for: Case Report: Pregnancy complicated with pulmonary arteriovenous malformation—diagnosis and surgical management
Source: Front Surg. 2025 Oct 8;12:1647557. doi: 10.3389/fsurg.2025.1647557 (PMC12540385; doi:10.3389/fsurg.2025.1647557)

**2023-04-19**

**Plain CT scan**


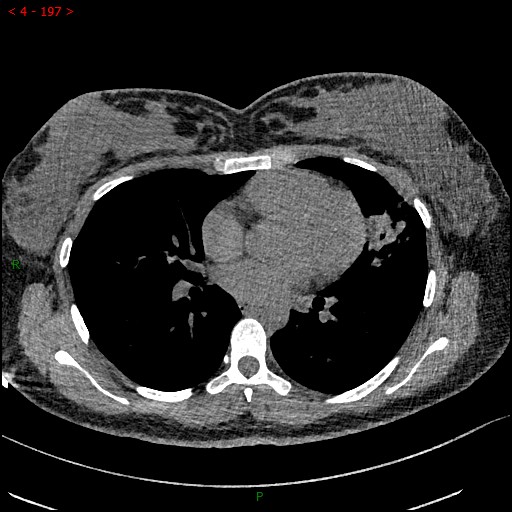

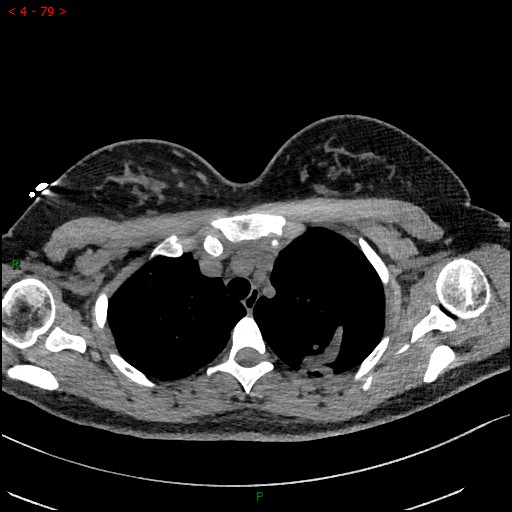

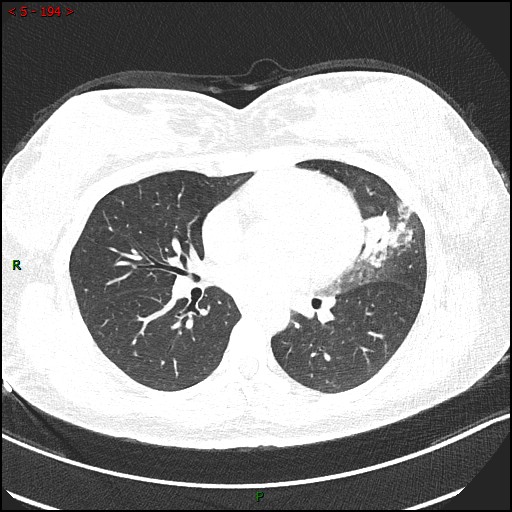

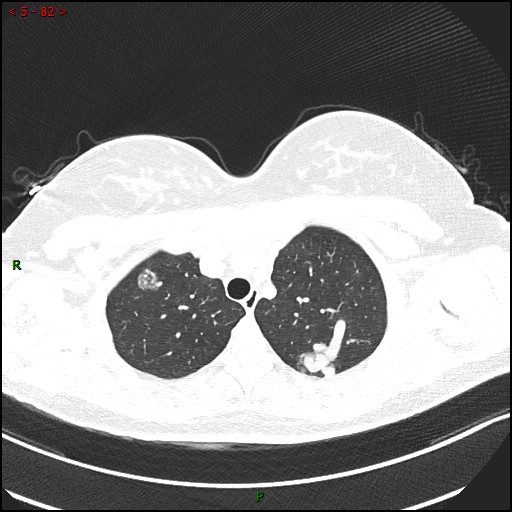


**contrast-enhanced CT imaging**

**Lesion in the apical segment of the left upper lobe**


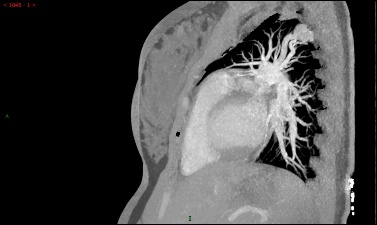

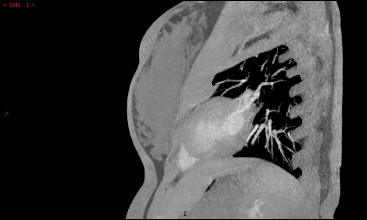

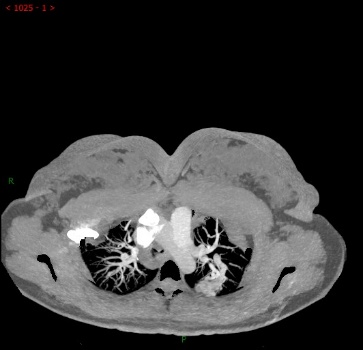

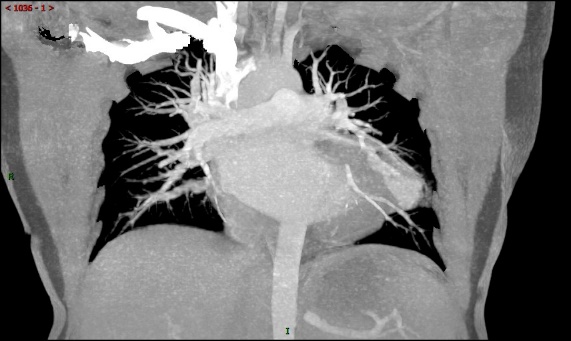

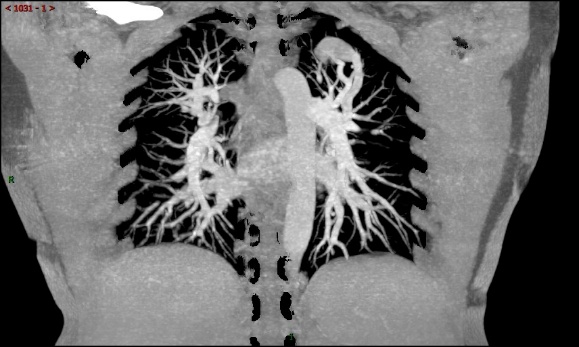


**Lesion in the lingular segment of the left upper lobe**


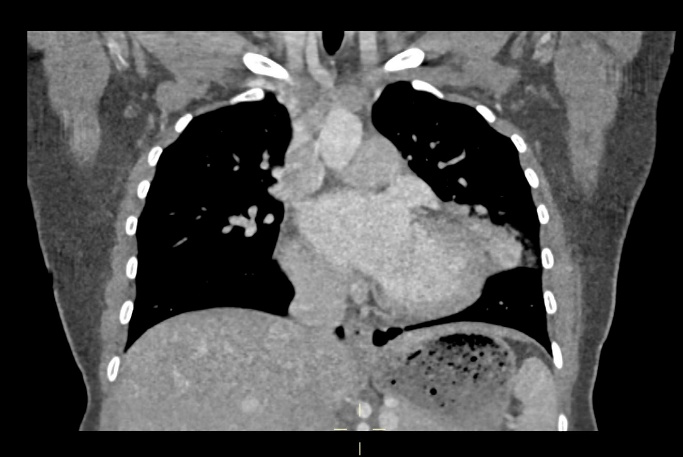

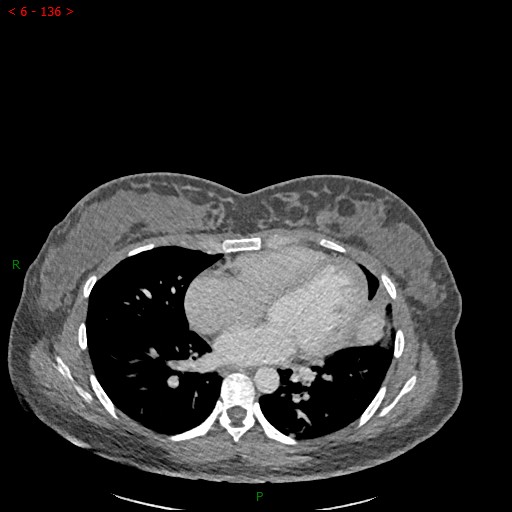


**2023-06-06**

**Pre-procedural phase before the first intervention**


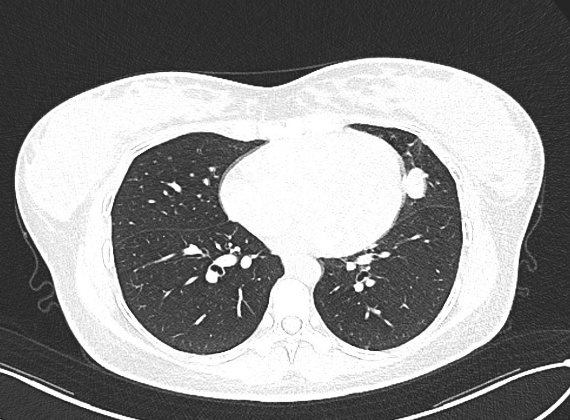

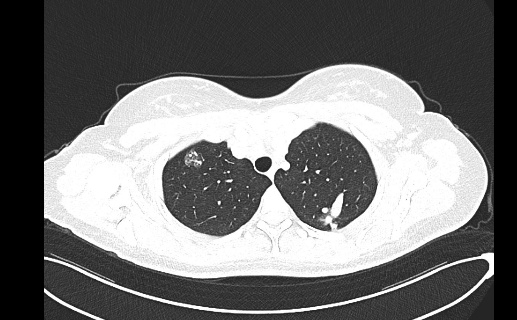

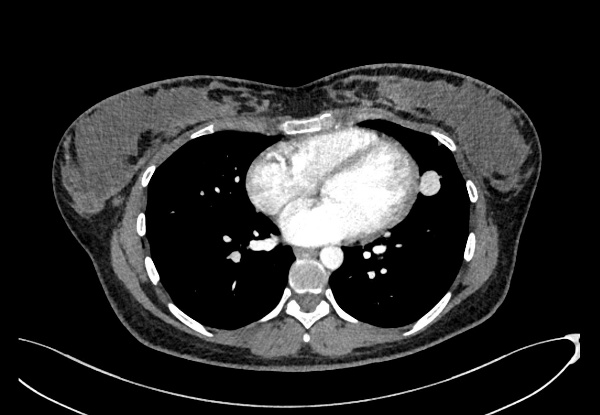

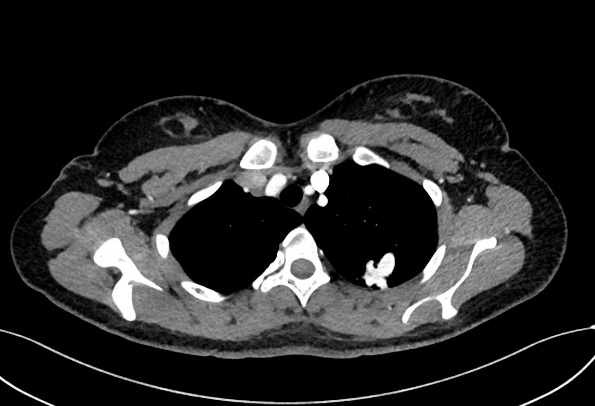


**2023-06-08**

**First interventional procedure**

**Embolization of the lesion in the lingular segment**


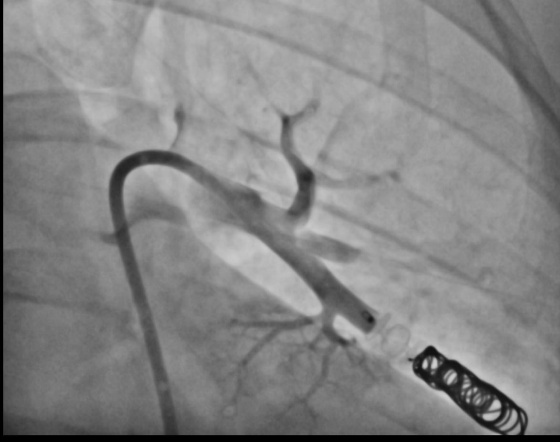

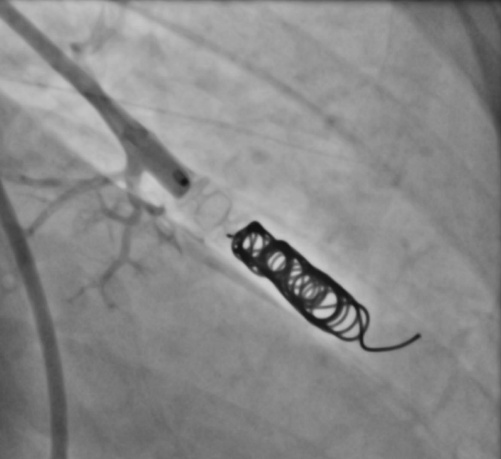

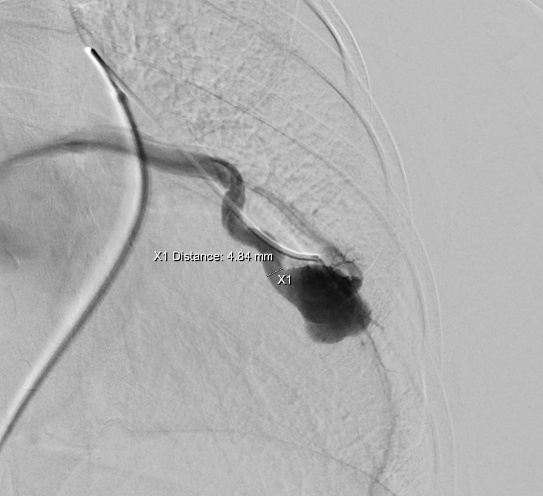

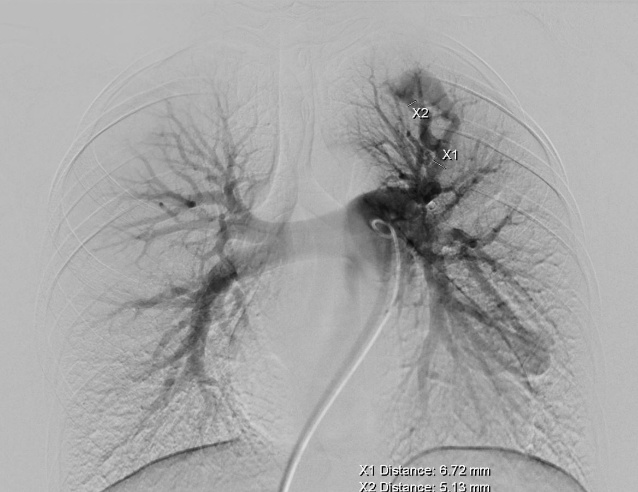


**2024-04-01**

**Re-examination**

**Embolization of the lesion in the lingular segment of the left lung was satisfactory, while the lesion in the apical segment of the left upper lobe remained unchanged.**


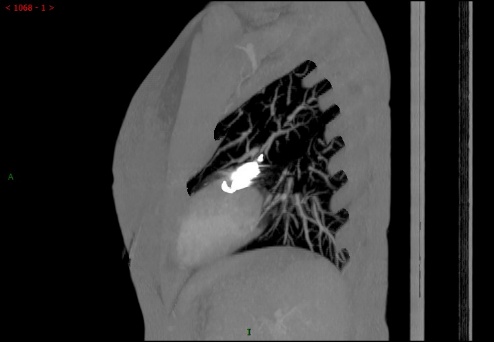

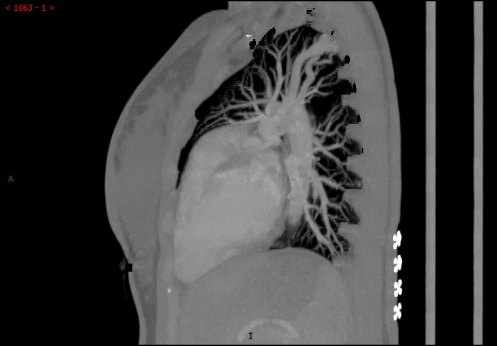

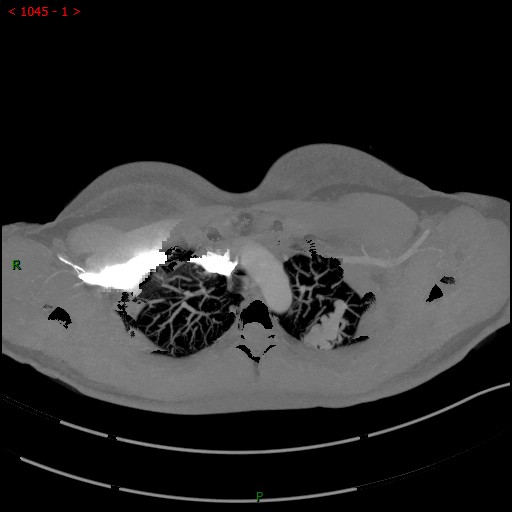

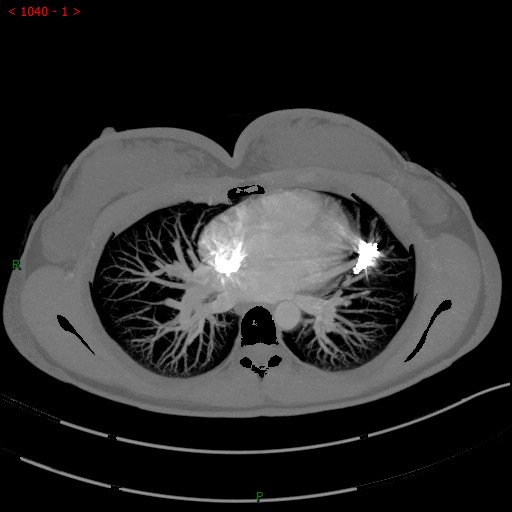

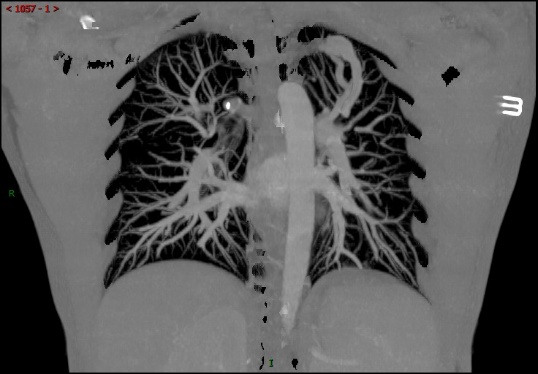

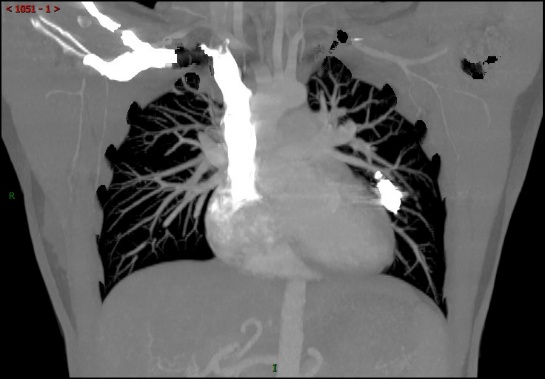


**2024-06-13**

**Second interventional procedure**

**Embolization of the apical segment**


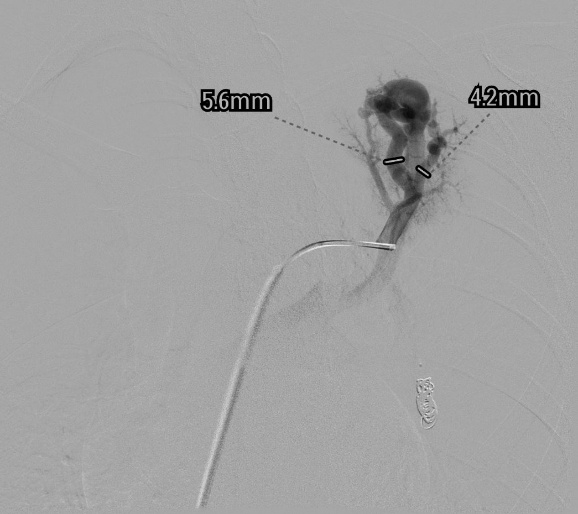

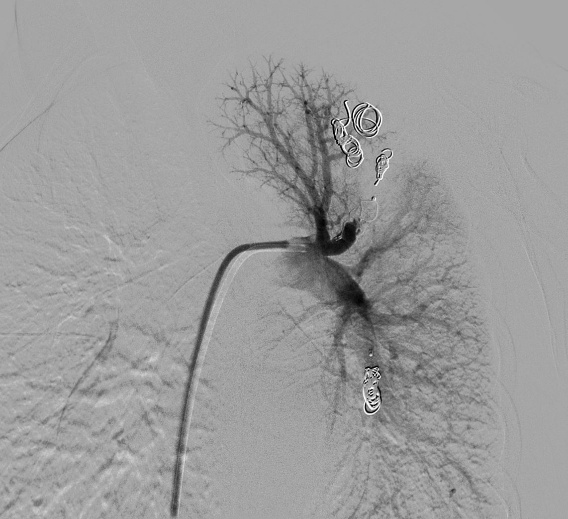

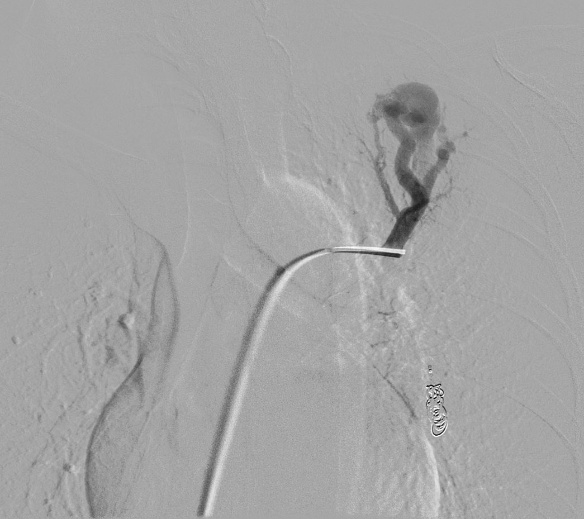

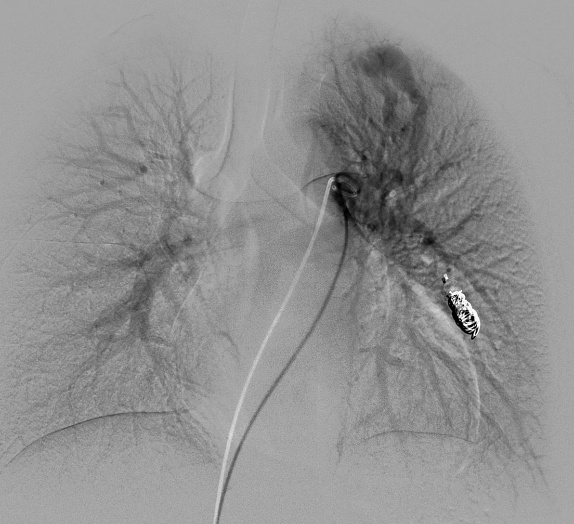

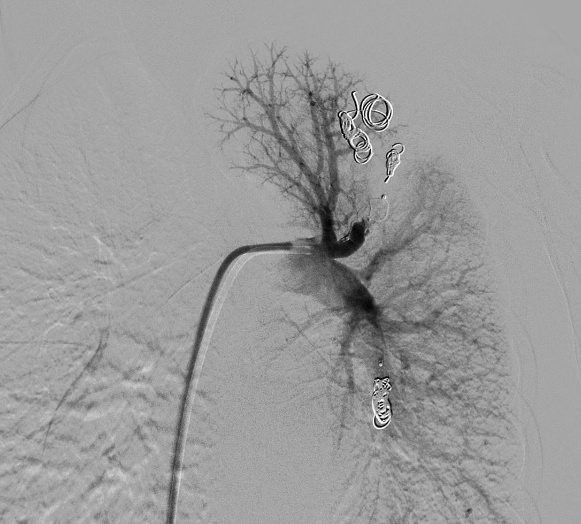


**2024-08-07**

**First follow-up examination**


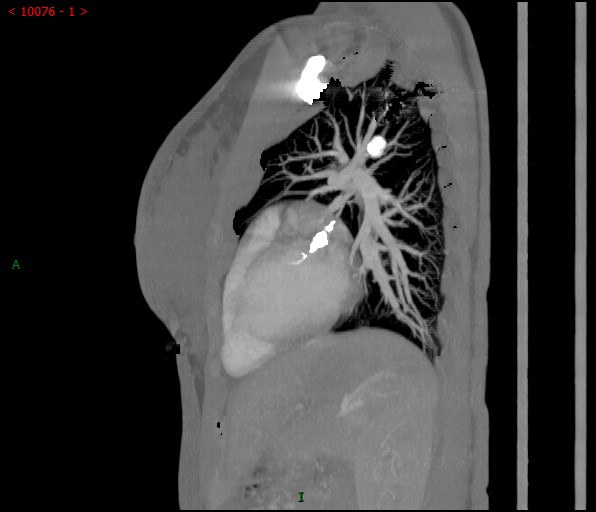

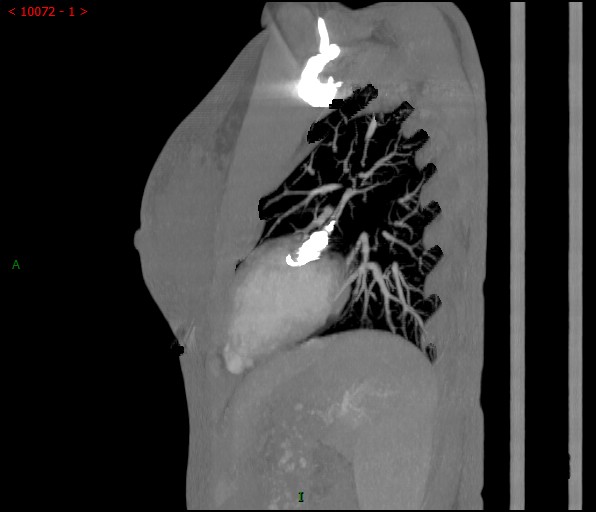

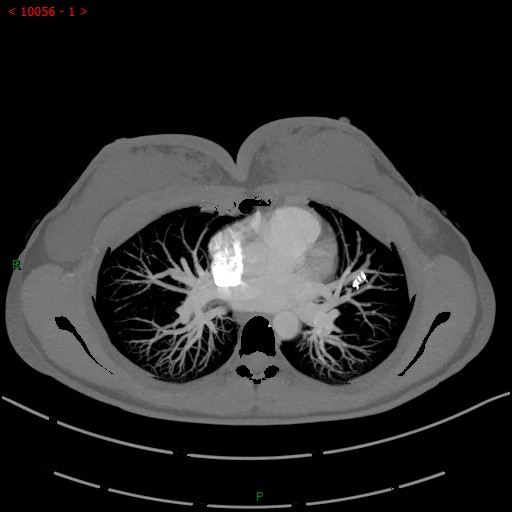

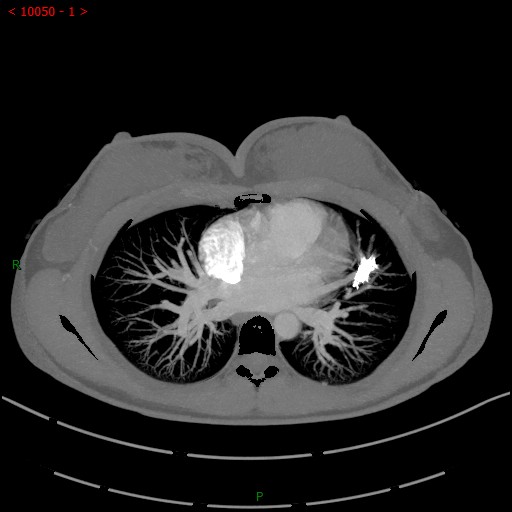

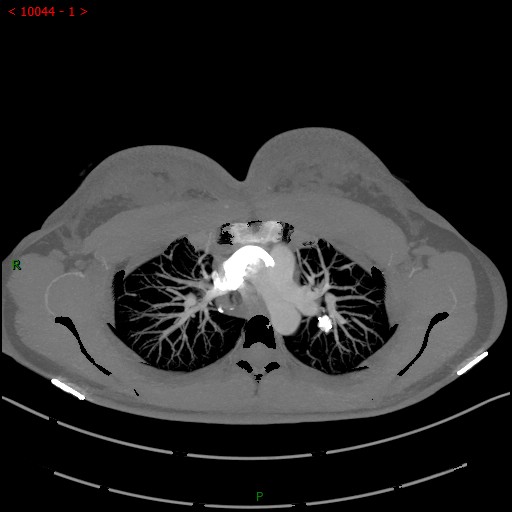

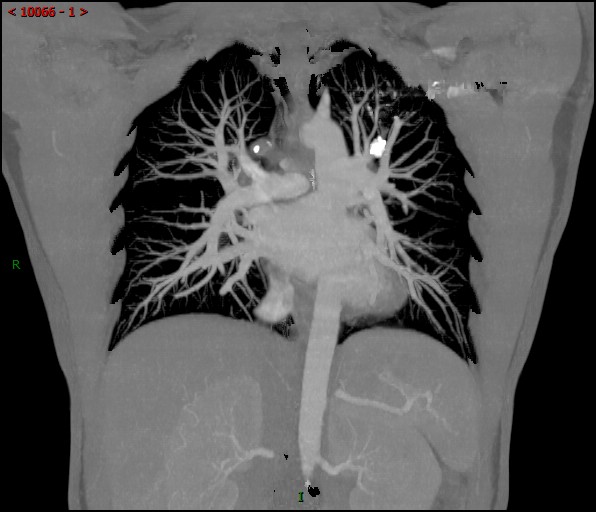

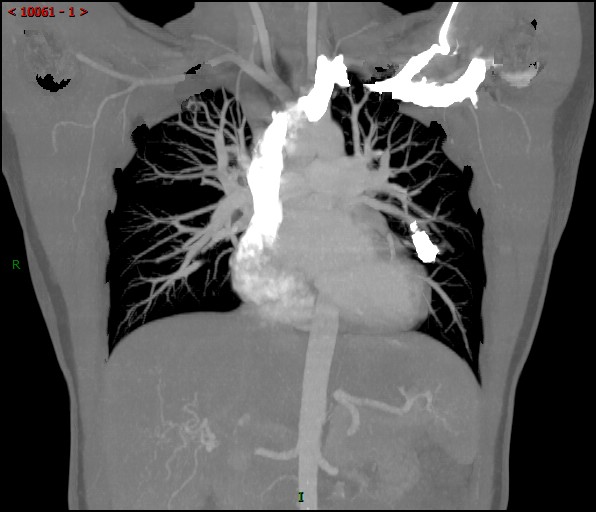

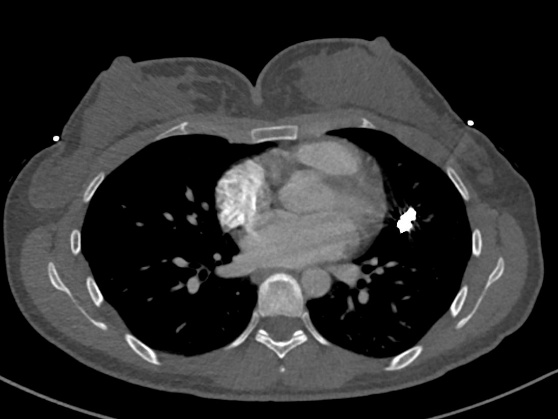

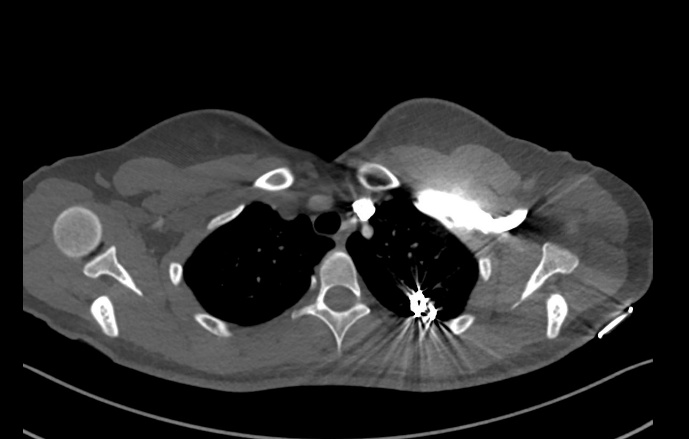

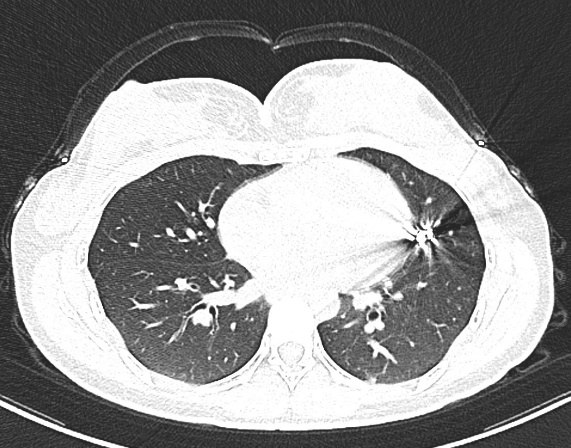

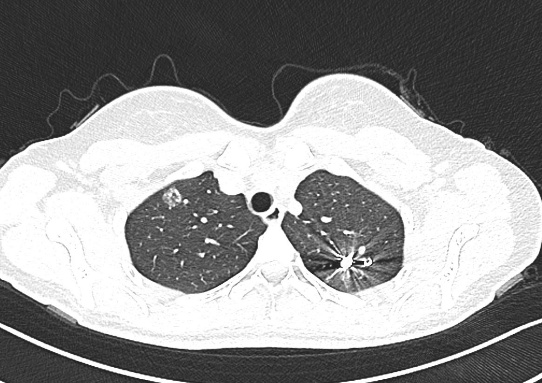


**2024-11-14**

**Second follow-up examination**


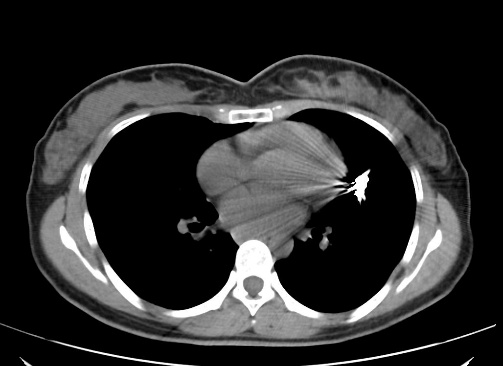

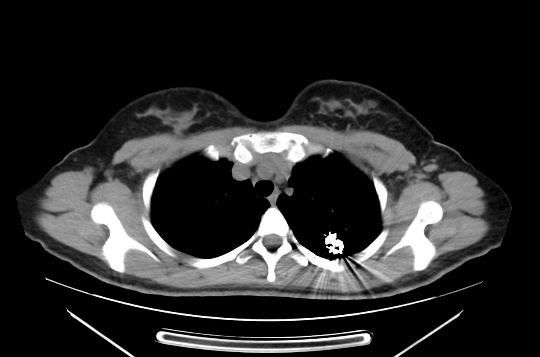

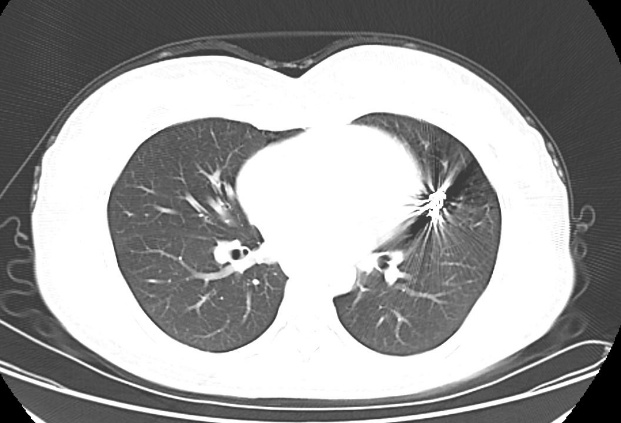

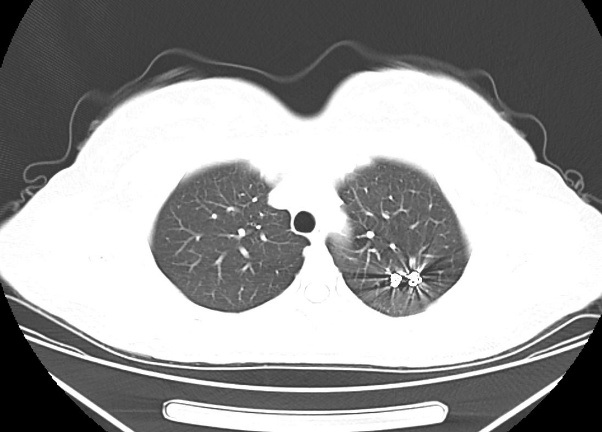

Supplement: Supplementary file 1 [file Supplementaryfile1.docx]
